# Supplementary material for: Pentraxin 3 Inhibits the Angiogenic Potential of Multiple Myeloma Cells
Source: Cancers (Basel). 2021 May 8;13(9):2255. doi: 10.3390/cancers13092255 (PMC8125855; doi:10.3390/cancers13092255)
Supplement: Supplementary file 1 [file cancers-13-02255-s001.zip › cancers-1190242_supplementary.pdf]

cancers

MDPI

Supplementary Materials

Pentraxin 3 Inhibits the Angiogenic Potential of Multiple Myeloma Cells

Roberto Ronca, Sara Taranto, Michela Corsini, Chiara Tobia, Cosetta Ravelli, Sara Rezzola, Mirella Belleri, Floriana De Cillis, Annamaria Cattaneo, Marco Presta and Arianna Giacomini

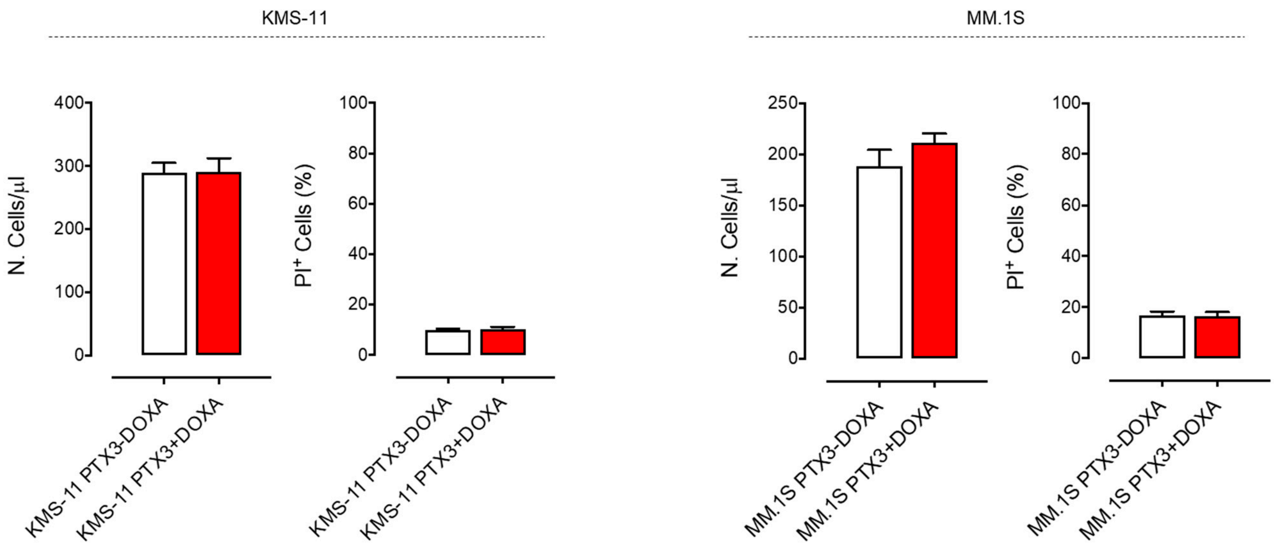

Figure S1. PTX3 does not affect MM cell proliferation and survival in standard cell culture conditions. Cytofluorimetric analysis of viable cell counting and cell death by Propidium Iodide staining (PI) 96 hours after cell culture in the presence or absence of DOXA.

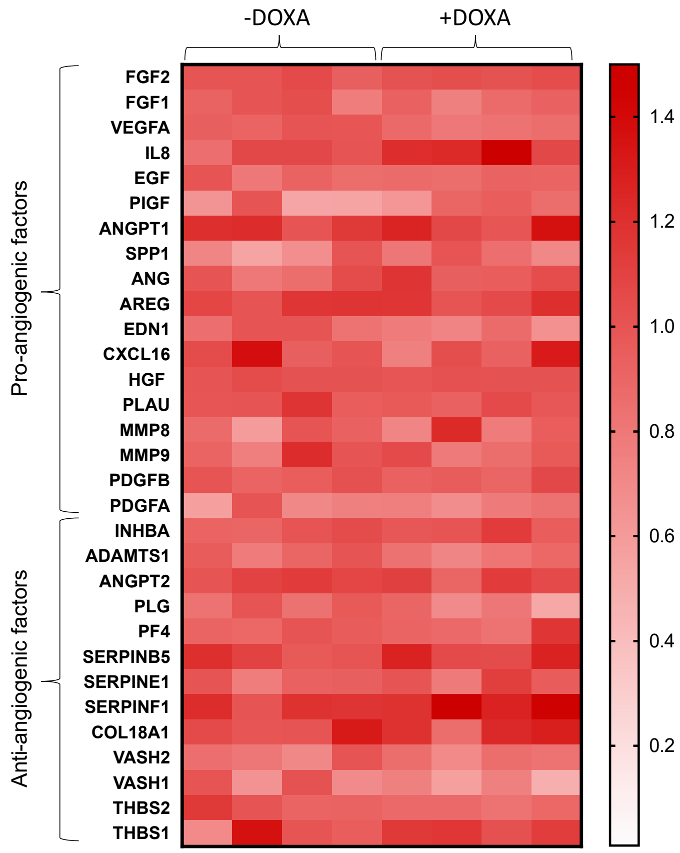

Figure S2. PTX3 does not affect the expression of pro-/anti-angiogenic factors. Gene expression profiling analysis of KMS-11 PTX3 cells induced (+DOXA) or not (-DOXA) to express PTX3. The analysis was performed in quadruplicate.

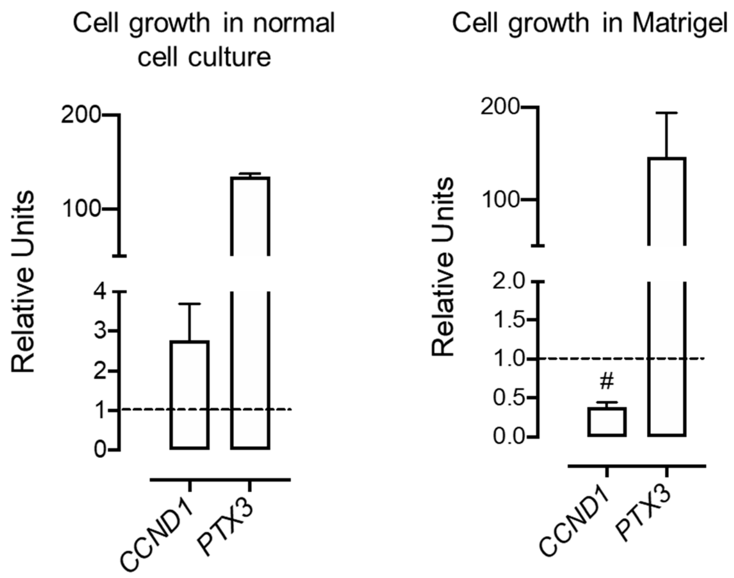

Figure S3. PTX3 affects MM cell proliferation when cells are grown embedded in Matrigel. qPCR analysis of *CCND1* and *PTX3* expression levels 96 hours after normal cell culture or when cells were grown in Matrigel in the presence of DOXA. Data are normalized to the expression levels measured in DOXA-untreated control samples (dashed line).
