# Supplementary figures and images for: Pentraxin 3 Inhibits the Angiogenic Potential of Multiple Myeloma Cells
Source: Cancers (Basel). 2021 May 8;13(9):2255. doi: 10.3390/cancers13092255 (PMC8125855; doi:10.3390/cancers13092255)

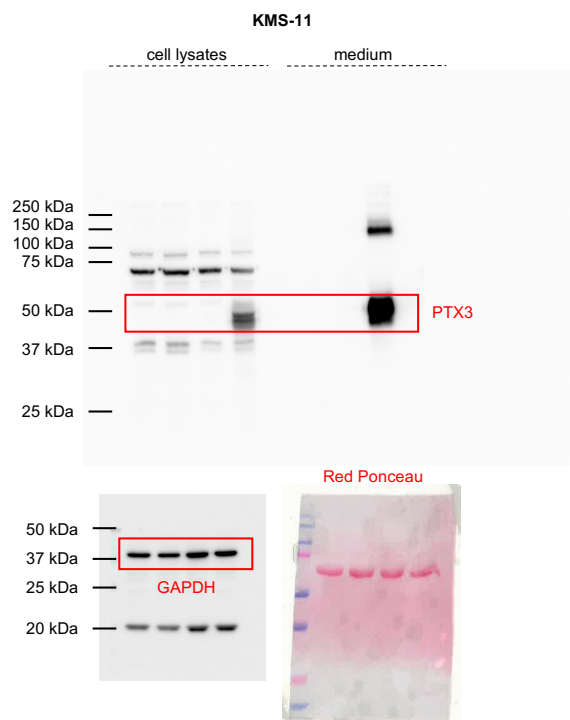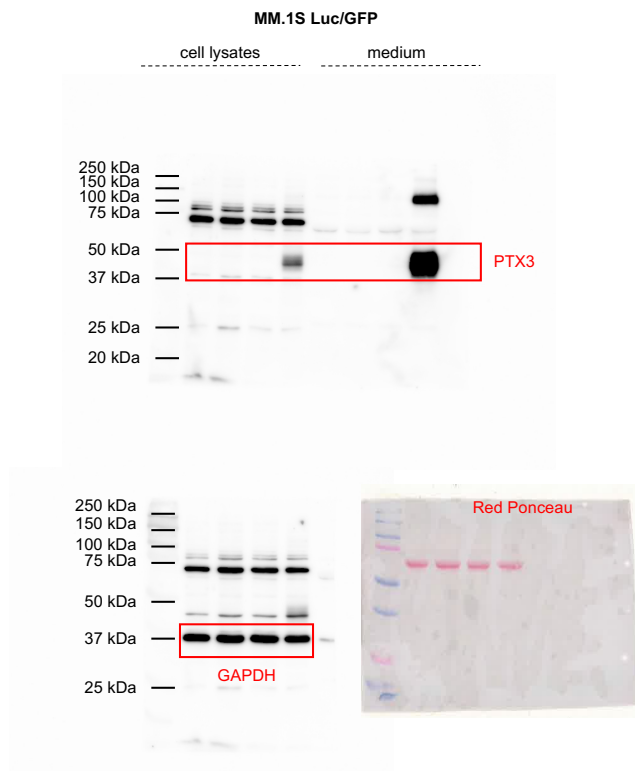

Supplement: Supplementary file 1 [file cancers-13-02255-s001.zip › cancers-1190242_western blot.pdf]
